# Post-consent assessment of dental subjects' understanding of informed consent in oral health research in Nigeria

**DOI:** 10.1186/1472-6939-10-11

**Published:** 2009-08-01

**Authors:** Olaniyi O Taiwo, Nancy Kass

**Affiliations:** 1 Intercountry Centre for Oral Health (ICOH) for Africa, Jos Plateau State, Nigeria; 2 Johns Hopkins Berman Institute of Bioethics, Johns Hopkins Bloomberg School of Public Health, Maryland USA

## Abstract

**Background:**

Research participants may not adequately understand the research in which they agree to enroll. This could be due to a myriad of factors. Such a missing link in the informed consent process contravenes the requirement for an "informed" consent prior to the commencement of research. This study assessed the post consent understanding of Nigerian study participants of the oral health research they were invited to join.

**Methods:**

A descriptive cross sectional study with research participants who had just consented to one of three ongoing research studies on oral health. Study sites included two centers, one in the northern and one in the southern part of Nigeria. Data were collected using a combination of quantitative and qualitative methods.

**Results:**

A total of 113 research participants were interviewed. The southern part of the country had 58 respondents with the north having 55. The age range was 21 – 80 years. Mean age was 46.1 (SD16.3). The sample was predominantly male (69.9%) and married (64.6%). There was poor understanding of some key elements of the informed consent process such as involvement in research, benefits, contacts, confidentiality and voluntariness. Some identified factors potentially compromising understanding were poverty, illiteracy, therapeutic misconception and confusion about the dual roles of the Dentist and the researcher.

**Conclusion:**

The participants recruited into the oral health research in Nigeria did not adequately understand the studies they were invited to join nor do they understand their rights as research participants. Measures should be taken to include research bioethics into the curricula of Dental schools and to train oral health researchers in the country on research ethics.

## Background

A key mechanism to improving the practice of health care and the health of populations is conducting research. In most developing countries, however, research regulations do not exist or are in their initial stages. [1] Indeed, in most developing countries, bioethics is a nascent area of study, and researchers often feel ill-equipped to analyze the ethics of a research project. [1] In Nigeria, The National Code for Health Ethics Research was only recently promulgated in 2007, a Code developed largely through adaptation of Codes from wealthier countries. The Nigerian Code, however, like other Codes of ethics, requires informed consent as a key component of ethically sound research practice.

Informed consent has been defined as "an autonomous authorization by individuals of a medical intervention or of involvement in research" [2]. To be valid, informed consent must include disclosure, understanding, voluntariness and competence [2]. These are built into the informed consent process to safeguard the interests of the research participant. Competence or capacity for decision making is composed of four functional abilities: the ability to *understand *relevant information; the ability to *appreciate *the nature of a situation and its likely consequences; the ability to *reason *through the information and weigh options logically; and the ability to *communicate *the choice [3,4]. Whether consent is oral or written, it may not necessarily be informed [5]. To determine if the consent is valid, one should ascertain whether participants actually understood the information provided and whether they believe they gave voluntary consent for the study [4].

Many studies reveal poor understanding by research participants about the research process. Indeed, some participants may not even be aware that they are participating in research [3,6]. Other deficiencies identified in patients' understanding include lack of understanding of randomization procedures and placebo treatments; poor recall of supplied information; inadequate recall of risks of procedures or treatments; lack of awareness of the ability to withdraw at any time; lack of awareness of alternative sources of care, and, significantly, "therapeutic misconception" whereby research is mistaken for medical care and/or there is confusion about the dual roles of physicians/researchers. [3,6-10].

Reasons highlighted as causing a reduction in participants' understanding of consent forms in particular include: information that is too sparse or too complicated and insufficient opportunity for participants to ask questions [9]. Informing patients in an effective, understandable way may also be compromised when the consent document is seen as a legal instrument rather than a communication tool [11] or when the patient has cognitive impairment [4].

In other cases, lack of understanding is not necessarily a result of an insufficient mechanism for supplying information. It may also be a result of lack of familiarity with research as an activity on the part of participants. In such cases, participants may interpret what researchers tell them through their own lens, assuming that the activity is, by definition, beneficial to them or is being offered to them because it is in their best interest to participate. [12] In some instances, participants may choose to disregard some of the information provided. This presents researchers with a problem because in many cases they cannot be certain why participants lack understanding [9]. The severity of a patient's illness, hope and optimism also can influence the extent to which patients accurately understand information in the consent form [6,13]. "Deep interpersonal trust in individual clinician researchers, and institutional trust in hospitals, in research institutions and in the research enterprise can also have a profound impact on the communication and understanding of health and research information, on ability to distinguish between medical care and research, and on perceptions of study benefits and risks'' [13].

Dentistry, like medicine, is a Hippocratic profession and is therefore committed to ongoing research into the causes and treatments of disease. It, too, must be bound by the same ethical standards required for medical research. Those standards include that informed consent must be obtained from individuals selected to participate in dental research [14]. While there has been a significant emphasis on informed consent in medical research, there has been less emphasis in the literature regarding informed consent and dental research. Further, while informed consent for medical research has become normative in the United States and other Northern countries, this is not the case throughout Africa. As such, we conducted a study to examine what dental patients involved in oral health research in Nigeria understand about their own participation. This study describes the post consent understanding in a group of Nigerian oral health research participants.

## Methods

### Study Design

A descriptive cross sectional study was used to conduct this investigation.

### Participants and Procedure

Respondents were selected by a nonrandom convenience sampling. Adults were eligible who had just provided consent (within the last hour) to one of three ongoing independent oral health research projects (called here "parent studies") and who were willing to undergo a post assessment interview. An adult was defined as someone aged 18 years and above. Two of the three parent study research projects had no IRB approval. According to parent study investigators, all studies solicited oral, rather than written consent (although its formality and completeness is unknown to us). Participants from the ongoing independent oral health research projects were not recruited as part of normal dental care nor were they to receive additional dental care after the respective studies. Two of the three parent studies took place in the neighboring communities to a Teaching Hospital while the third took place in a Primary Health Care center having no dental facility. The principal investigators of the parent studies (who are Nigerian dental researchers) informed the participants about the assessment interview for the present study on consent understanding. If the respondent was interested, he or she was referred to the investigator for this consent project (OOT) to complete the consent process and to complete the interview. This was done individually in the participant's local dialect for those who do not understand English. No staff of the parent studies (independent oral health research projects) was directly or indirectly associated with this consent study. The purpose and objectives of the parent studies were discussed with the investigator (OOT) prior to the commencement of data collection to help ascertain the authenticity of the response of the respondents due to the generic nature of the data collection instruments.

### Study Sites

The study was conducted in two Nigerian states – one in the Northern and one in the Southern part of Nigeria. The commonest local language spoken in Northern Nigeria is Hausa while in the particular State used in the South, it is Yoruba. There were two parent studies included from the southern part of the country and a single study from the northern part. To protect the identity of the studies, we will refer to the two studies in the southern part of Nigeria as studies A and B and the study in the northern part as study C. All three of the parent studies were cross sectional in nature requiring just a single contact with the patient. Study A required only a questionnaire to be administered to oral health patients, while studies B and C required both a questionnaire and a detailed examination of the oral cavity of participants. None of the studies involved more than a minimal risk of harm. Studies A and B took place in the neighboring communities to the Teaching Hospital while study C took place in a Primary Health Care center.

### Data Collection Instruments

Data collection for this study was completed using a quantitative survey. The variables of interest include knowledge of being in a research study, understanding of the purpose of the parent study, risks, benefits, confidentiality, voluntariness, and whom to contact in case of any questions. The quantitative survey was administered by an interviewer. The survey was developed from the expected components of an informed consent form as specified in the US federal regulations. About 80% of the survey questions were taken from previously designed informed consent assessment tools; the Quality of Informed Consent (QuIC) questionnaire [6] and the Deaconess Informed Consent Comprehension Test (DICCT).[15] The survey was anonymous.

A semi – structured qualitative interview (which is centered on the understanding of research purpose and benefits) was also conducted with 8 participants who were purposely selected based on their readiness to do the interview. This interview was conducted to shed more light on the quantitative assessment. All interviews were conducted by OOT.

### Analysis

Completed surveys were examined for completeness before data were entered into SPSS^® ^14.0. The data were summarized using descriptive statistics including frequency distribution, measures of central tendency and dispersion.

Qualitative interviews were transcribed. The transcripts were manually reviewed to check for themes which shed more light on the topic of understanding

### Research Ethics

The study was reviewed and approved by the Institutional Review Board of the Johns Hopkins University Bloomberg School of Public Health in the United States as well as by the local Institutional Review Boards of the University Teaching Hospitals at the study sites.

## Results

### Participant Characteristics

A total of 113 research participants completed the surveys. Studies A and B (Southern part of the country) had 17(15%) and 41(36.3%) respondents respectively while study C had 55 (48.7%) respondents. The age range was 21 – 80 years. Mean age was 46.1 (SD16.3). The majority of the sample was male (69.9%) and married (64.6%). There were twelve respondents (10.6%) who had attained a form of tertiary education. Table 1 summarizes the sample's demographic characteristics.

**Table 1:** Sample Characteristics from the Northern and Southern part of Nigeria

**Characteristics**	**South** **N (%)**	**North** **N (%)**	**Total**
Number (% of total)	58 (51.3)	55 (48.7)	**113**
Age range (in years) (SD)	21 – 58 (8.45)	50 – 80 (8.08)	**21 – 80 (16.3)**
**Gender**			
Male	50 (86.2)	29 (52.7)	**79**
Female	8 (13.8)	26 (47.3)	**34**
**Highest Education Level completed**			
None	1 (1.7)	36 (65.5)	**37**
Primary	19 (32.8)	12 (21.8)	**31**
Secondary	32 (55.2)	1 (1.8)	**33**
Tertiary	6 (10.3)	6 (10.9)	**12**
**Marital Status**			
Single	29 (50)	-	**29**
Married	26 (44.8)	47 (85.5)	**73**
Divorced	-	-	-
Separated	1 (1.7)	-	**1**
Widowed	2 (3.4)	8 (14.5)	**10**
			
**Prior participation in Oral Health Research**			
Yes	5 (8.6)	1 (1.8)	**6**
No	53 (91.4)	53 (96.4)	**106**
Not sure	-	1 (1.8)	**1**

### Knowledge of and involvement in Research

Fifty – two (46%) respondents said they had no idea what research or experimental study in dentistry connotes. Twenty – three percent said research was learning new things while 5.3% thought it was another treatment. Other responses included *checking out the health of people, asking questions, finding out the known and the unknown, investigations and examinations*. Nearly all the respondents (105, 92.9%) said they did not know they were in a research study on their dental care The major reasons reported for why they were participating in the activity included to seek dental care (38.9%) and for free dental test/examinations (31%). Furthermore, they believed that the activity was been carried out to help people (39.8%), to address a research question (15%) and to find a new treatment (3.5%). Thirty – seven respondents did not know why the study was being conducted. Only 10 (8.8%) respondents knew that the study required just a single contact with them. Ninety – eight (86.7%) respondents did not know of their duration of participation in the study.

### Foreseeable Risks or Discomforts

Despite the fact that none of the studies posed any risk or discomfort more than minimal risk, 70.8% of the respondents did not know if there was any risk attendant with their participation in the study. Only one participant cited that the procedure might be inconvenient.

### Benefits

For the studies (A, B and C), there is no direct benefit to the participants. The only benefits available are indirect benefits, also known as collateral benefits, which accrue to the participants simply by being in research [8]. These include referral and oral health counseling that likely would have been made after participation in any of the three studies, particularly the two that examined the oral cavity. Twenty-six respondents understood the benefits of the study. Twenty-five (22.1%) were not sure of the benefits they would get from the study. Other (incorrect) benefits mentioned in the qualitative interview by two respondents included the expectation or *the provision of free drugs *and *attending to their dental health*. Another benefit mentioned by a 55 year old man was; "*the less privileged people would have the opportunity to have free treatment since they can't go to the hospital or afford their fares." *About half (49.6%) of the respondents thought their participation in the study would be beneficial to other people in the larger society. All the respondents with tertiary education (14) were positive that their participation in the research would benefit other people. Approximately one-third of respondents believed they (the participants) were the only ones benefiting while the others were not sure if other people would be benefiting through their participation.

### Contacts and Confidentiality

Fifty – two (46%) respondents did not know whom to contact if they had any questions about the consent process or the study. Most of the respondents (85.8%) were not aware of how their records would be kept. Three respondents believed records would be kept with the hospital records and 13 others said they would be kept in the researcher's office.

### Voluntariness

Nearly all the respondents, (93.8%) claimed they were not asked if they wanted to join the study though 8 (7.1%) respondents knew it was a research. When asked if they knew they had a choice to join or not, 17 (15%) said Yes, 80.5% said No while 5 (4.4%) respondents were not sure. The main reasons why the research respondents decided to partake in the study was that they thought it offered them free treatment (70%). Some said they just felt like participating (9.7%) while 2 respondents said they were encouraged by the Doctor. One respondent, a 52 year old (male) University graduate who is a farmer, said during the qualitative interview: "*As an educated man in the community and also the secretary of the community council, if I refuse to participate, others might also refuse because they expect me to understand what is going on"*. Also, 10 respondents of the 14 with tertiary education knew they could withdraw their participation at any time. One respondent said she was participating because she was not feeling well and another added in the qualitative interview that: "*I also conduct research so there is no problem with this. I chose to partake so that I can see the end"*. When asked if they believed they could stop participation in the study at any time, 85.8% said No, 12.4% said Yes while 1.8% of the respondents were not sure if they could stop participation (Table 2).

**Table 2:** What would happen if you chose to stop your participation at any time?

**Response**	**N**	**%**
Nothing would happen	34	30.1
My Doctor (the researcher) would not be happy with me	10	8.8
I don't know	8	7.1
Could be a bad decision for me clinically	38	33.6
Others	23	20.4
*"I might regret not utilizing the opportunity for the free treatment"*		
*"How can I stop participation when I have come to seek medical help?"*		
*"I would discourage others who want to join"*		
*"I would spoil their work."*		
*"It is compulsory"*		
*"It shows that there is no reason for my coming"*		
*"It is a research work, I can't stop"*		
*"I would be insulting the Doctors by stopping"*		
*"I would have cheated myself"*		
*"It shows that I'm not serious"*		
*"I can't stop because Doctor is doing something good"*		
*"I would go back with the problem I came with"*		
*"Doctor would go away"*		
*"I can't say I would stop participation since I chose to come for my health sake"*		
*"My conscience would not allow me because it is helpful"*		
*"I want to know what they are doing" etc*		

## Discussion

The results of this investigation suggest that the respondents had a poor overall understanding of the research programme in which they had enrolled. This calls to question whether they were adequately informed in the consent process – or indeed whether much of a consent process had been conducted at all. Given that two of the studies had not gone through an IRB, and all three used oral consent, it is reasonable to speculate that a rather informal and, perhaps, superficial discussion of study participation had been provided by investigators who may have received little training in research ethics. That participants have limited understanding is, however, consistent with several studies conducted in other parts of the world. [10]

The compromised understanding we observed might have been exacerbated as a result of language barriers. [16] For example, the word "research" does not have a corresponding term in the local languages of Northern and Southern Nigeria. The common lingua franca in the southern part where the study was conducted is Yoruba and the closest Yoruba words to research are "*iwadi*" or *"ayewo" *which actually mean investigation or examination. In the northern part of the country, the commonest language spoken apart from English (for the literate populace) is Hausa. Likewise, the closest word to research in Hausa is "*binchike" *which can also be translated to mean investigation. Securing an informed consent with research translated into these words brought a new meaning to the word, potentially complicating the consent process as well as our assessment of it through our own interviewing. This terminology problem has also been reported in a study conducted in Kenya [17]. In addition to these specific challenges, informed consent research often faces the challenge of good communication in settings where investigators have significantly different educational and socio-cultural backgrounds to study participants [17].

Education is an important factor which we believe can enhance how investigators execute the informed consent process. While some sort of research practicum is generally required for most health professional training in Africa, parallel training in the ethics of human subjects' research is not the norm. Respondents, similarly, may not have been exposed to the concept of research unless they had heard of it through their own education or training. Other studies had reported the effect of the educational level of the respondents in the understanding of the informed consent process [3].

A person in a poor developing country can be told that this is research, but it may be that what they hear is that they have a chance to get health care or else refuse their only good chance at care. Most of the respondents thought that the parent oral health research was an outreach program for free oral health service. This may be because of the gradually increasing frequency of such services being provided by some non – governmental organizations (NGOs) and political appointees in Nigeria. A lot of this free medical outreach is conducted with foreign support. As such, the presence of a group of health care workers in a locality is usually symbolic of free treatment unless proven otherwise. Thus, that respondents surmised they were receiving free care, even when no one articulated that, might be considered a logical deduction. As such, it is not rational for participants to think about the idea of voluntary withdrawal. The perception that they were to receive some free care would have been an incentive to join and to stay enrolled, particularly among a fairly impoverished population. Actually, some of the respondents were visibly excited about the (erroneous) preconceived opportunity to have their ailments addressed. Any activity by medical personnel in the community is expected to be therapeutically beneficial. This also is consistent with studies that had highlighted therapeutic misconception as an area of misunderstanding in the informed consent process [8]. Most people are yet to differentiate the dual role of the professional as both a doctor and a researcher and these beliefs may be particularly prevalent where research literacy is minimal. While this confusion is more understandable in the two dental studies that performed an oral examination as part of the research, it is particularly troubling for the study that only involved a questionnaire. Professionals working in these settings, aware of the likelihood of these perceptions, should take care to emphasize the quite different nature of a research inquiry, particularly those that are descriptive or observation and provide no clinical interventions.

For the three studies we assessed, all three investigators reported informally to us that they had secured oral consent from the participants before involvement in the study. In the process of securing consent, however, fatigue might have set in on the part of the person securing consent for repeating it over and over. It is also possible that the consent did not contain all of the relevant constituents of the informed consent process outlined in international codes and national regulations. Anecdotally, and in our experience, it seems that oral health researchers in Nigeria have had less exposure to training opportunities in research ethics than their medical counterparts. The level of knowledge of the researcher about research ethics would determine the extent to which its guidelines would be observed for research involving human participants. That none of the respondents mentioned certain elements of consent, such as asking for involvement in research, whom to contact, voluntariness and description of possible risks and/or discomfort, suggests that investigators may not have included these at all in the informed consent process.

The average Nigerian, like those in other developing countries, sees the doctor as a man/woman of knowledge whose views are to be followed. "The motives of the professional's conduct are not questioned or even considered; they are supposed to be almost divine, in accordance with the authority he or she possesses". [1] This has kept the medical/dental profession paternalistic with patients unlikely to question their judgments or views. When the health-care professional asks the patient to participate in a research study, these cultural beliefs lead to almost uniform agreement. [1] It may be considered dishonoring to query the views of the doctor, exemplified by the two respondents who said that refusal to continue participation in the study was tantamount to insulting the doctor or that the doctor would go away.

There were several limitations to this study. First, it involved only three studies in two Centers in Nigeria. There are five more Centers in the country where this study could have been conducted. Further, all of these studies were minimal risk, all involved single encounters, and all used oral consent. It may be that for higher risk studies, more care is taken to make sure that participants understand what the research is about, or it may be that in studies with written consent, participants have fuller understanding. Another limitation of the study is that the details of the parent studies' consent procedures and content were reported to us informally by parent study investigators, but these procedures were not validated by us through direct observation. As such, we are unable to determine whether limitations in understanding we documented from our respondents were the result of inadequate provision of information, or misunderstanding of information by the respondents, or both. Also, these findings could have been due to the survey instrument used which was largely adapted from those used in western countries. It contained some words whose translations in the local languages may not have appropriately conveyed their intended meaning. Nonetheless, this study provides a snapshot of what participants understand, having received whatever is the current approach for consent, in oral health research in two centers in Nigeria.

## Conclusion

This study demonstrates that currently, at least some dental research participants in Nigeria do not have an adequate understanding of the research in which they are enrolled. In our experience, very little training has been provided to dental researchers in Nigeria up to this point concerning what is required with regard to research ethics generally, and informed consent in particular. It is the tradition in Nigerian society to see the doctor as a person of understanding and authority which encourages a paternalistic relationship. Additional discussion with potential participants may greatly increase the likelihood that participants will understand research better in the future [18].

Given that investigators revealed informally that two of the three oral health parent studies had never undergone IRB review, it may be appropriate to formally survey more oral health researches in Nigeria in the future to determine how widespread such practices are. Further, if ethics review were required routinely of all research with human participants, it is likely that standard requirements such as adequate informed consent might become more normative in these settings.

## Competing interests

The authors declare that they have no competing interests.

## Authors' contributions

NK did the overall supervision. Both OOT and NK participated in the conception of the idea, designing the study, writing the protocol, preparation of the questionnaire and writing of the manuscript. OOT collected the data, entered it and performed the analysis.

## Pre-publication history

The pre-publication history for this paper can be accessed here:



## References

[B1] Oguze NY (2003). Research ethics committees in developing countries and informed consent: With special reference to Turkey. J Lab Clin Med.

[B2] Hyder AA, Wali SA (2006). Informed consent and collaborative perspectives from the developing world. Dev World Bioeth.

[B3] Dunn LB, Jeste DV (2001). Enhancing informed consent for research and treatment. Neuropsychopharmacology.

[B4] Wendler D (2004). Can we ensure that all research subjects give valid consent?. Arch Intern Med.

[B5] Dube-Baril C (2004). The personalized consent form: an optional but useful tool. J Can Dent Assoc.

[B6] Joffe S, Cook EF, Cleary PD, Clark JW, Weeks JC (2001). Quality of informed consent: a measure of understanding among research subjects. J Natl Cancer Inst.

[B7] Moodley K, Pather M, Myer L (2005). Informed consent and participant perceptions of influenza vaccine trials in South Africa. J Med Ethics.

[B8] Sugarman J, Lavori PW, Boeger M, Cain C, Edson R, Morrison V, Yeh SS (2005). Evaluating the quality of informed consent. Clin Trials.

[B9] Helgesson G, Ludvigsson J, Gustafsson S (2005). How to handle informed consent in longitudinal studies when participants have a limited understanding of the study. J Med Ethics.

[B10] Krosin MT, Klitzman R, Levin B, Cheng J, Ranney ML (2006). Problems in comprehension of informed consent and peri-urban Mali, West Africa. Clin Trials.

[B11] Sharp MS (2004). Consent documents for oncology trials: Does anybody read these things?. Am J Clin Oncol.

[B12] Featherstone K, Donovan JL (2002). "Why don't they just tell me straight, why allocate it?" The struggle to make sense of participating in a randomized controlled trial. Soc Sci Med.

[B13] Molyneux CS, Peshu N, Marsh K (2005). Trust and Informed consent: Insight from Community members on the Kenyan coast. Soc Sci Med.

[B14] Gillett GR (1994). Ethics and dental research. J Dent Res.

[B15] Miller CK, O'Donnell DC, Searight HR, Barbarash RA (1996). The Deaconess Informed Consent Comprehension Test: An Assessment Tool for Clinical Research Subjects. Pharmacotherapy.

[B16] Kusec S, Oreskovic S, Skegro M (2006). Improving comprehension of informed consent. Patient Educ and Couns.

[B17] Molyneux CS, Peshu N, Marsh K (2004). Understanding of informed consent in a low-income setting: three case studies from the Kenyan coast. Soc Sci Med.

[B18] Emanuel EJ, Currie XE, Herman A (2005). Undue inducement in clinical research in developing countries: is it a worry?. Lancet.

